# Reliability and accuracy of straightforward measurements for liver volume determination in ultrasound and computed tomography compared to real volumetry

**DOI:** 10.1038/s41598-022-16736-9

**Published:** 2022-07-21

**Authors:** D. Seppelt, M. L. Kromrey, T. Ittermann, C. Kolb, A. Haubold, N. Kampfrath, D. Fedders, P. Heiss, S. Hoberück, R. T. Hoffmann, J. P. Kühn

**Affiliations:** 1 Institute and Policlinic for Diagnostic and Interventional Radiology, University Hospital Carl-Gustav-Carus, TU Dresden, Fetscherstrasse 74, 01307 Dresden, Germany; 2 Department of Diagnostic Radiology and Neuroradiology, University Medicine Greifswald, Greifswald, Germany; 3 Institute for Community Medicine, University Medicine Greifswald, Greifswald, Germany; 4 Medical Department I, University Hospital Carl-Gustav-Carus, TU, Dresden, Germany; 5 Department of Diagnostic Radiology, University Medical Center Regensburg, Regensburg, Germany; 6 Department of Nuclear Medicine, Dresden University Hospital, Dresden, Germany

**Keywords:** Pathology, Liver diseases

## Abstract

To evaluate the suitability of volume index measurement (VI) by either ultrasound (US) or computed tomography (CT) for the assessment of liver volume. Fifty-nine patients, 21 women, with a mean age of 66.8 ± 12.6 years underwent US of the liver followed immediately by abdominal CT. In US and CT imaging dorsoventral, mediolateral and craniocaudal liver diameters in their maximum extensions were assessed by two observers. VI was calculated by multiplication of the diameters divided by a constant (3.6). The liver volume determined by a manual segmentation in CT (“true liver volume”) served as gold standard. True liver volume and calculated VI determined by US and CT were compared using Bland–Altman analysis. Mean differences of VI between observers were − 34.7% (− 90.1%; 20.7%) for the US-based and 1.1% (− 16.1%; 18.2%) for the CT-based technique, respectively. Liver volumes determined by semi-automated segmentation, US-based VI and CT-based VI, were as follows: 1.500 ± 347cm^3^; 863 ± 371cm^3^; 1.509 ± 432cm^3^. Results showed a great discrepancy between US-based VI and true liver volume with a mean bias of 58.3 ± 66.9%, and high agreement between CT-based VI and true liver volume with a low mean difference of 4.4 ± 28.3%. Volume index based on CT diameters is a reliable, fast and simple approach for estimating liver volume and can therefore be recommended for clinical practice. The usage of US-based volume index for assessment of liver volume should not be used due to its low accuracy of US in measurement of liver diameters.

## Introduction

An accurate assessment of liver size and volume plays an important role in the clinical routine. The actual size of the liver correlates very well with body size and lifestyle factors like alcohol consumption^1^. An increase in liver size constitutes a decisive indicator for different liver pathologies like fatty liver disease or steatohepatitis. In addition, monitoring the course of liver size is used for the evaluation of treatment success^2,3^. This may in particular play a role in the evaluation of the course of disease of infections such as mononucleosis^4^ or in the evaluation of changes in the context of dietary food conversions^5^. Estimating liver volume is also crucial for effective surgical planning^6,7^, necessary for interventional therapy, e.g. selective internal radiation therapy (SIRT), as well as for the evaluation of liver hypertrophy after portal vein embolization or split liver approach. In addition, the liver size and its change can be used for preoperative monitoring to reduce the risk of liver failure and postoperative complications^8^.

There are several methods available for assessing liver size. Although in clinical routine examinations liver size is approximated by percussion and auscultation, these techniques do not provide reliable information about the actual liver volume^9,10^ with nearly half of normal sized livers classified as enlarged and vice versa^11^. Apart from these unreliable clinical investigations, the size of the liver can be estimated by imaging techniques like ultrasound (US), computed tomography (CT) and magnetic resonance imaging (MRI). Their availability, costs and investigation times vary notably.

To date accuracy of volumetry of the liver using cross-sectional imaging has been validated in many studies^12,13^ and it is seen as gold standard for liver volume assessment. However, aside from the investigation itself, the volumetric post-processing with often manually performed organ segmentation is time-consuming and the necessary technical equipment is frequently not available^14^.

As an alternative to liver volumetry, volume indices based on simple maximum diameter measurements in all three planes on cross-sectional imaging can estimate the liver size^15^. Previous investigations demonstrated an excellent agreement between VI determined by MRI and true liver volume^15^. Likewise, ultrasound, as a fast and inexpensive method with real-time imaging and high availability in clinical settings, may also be suited for evaluation of liver diameters, especially if frequent controls at short intervals are necessary to monitor the development of liver changes.

Therefore, the purpose of this study was to investigate the suitability of volume indices based on either US or CT to determine liver volume in clinical routine.

## Material and methods

### Study population

Between June 2017 and August 2018, 66 patients (38 men and 22 women, mean age 66.8 ± 12.6 years, range 19–88 years, 6 exclusions) underwent a clinically indicated CT scan of the liver as well as ultrasonography.

The Ethics Committee of the University of Dresden approved the prospective study and it conforms to the Declaration of Helsinki. Written informed consent was obtained from all study participants.

The decisive inclusion criterion was a complete image of the liver within an examination phase of the CT scan. Patients were not selected at baseline for morphological or anatomical liver changes in order to achieve the greatest possible variation in liver size in the study group. One exclusion criterion for patients was poor conditions for ultrasound examination, such as inadequate inspiration compliance, which resulted in the edges of the liver not being fully visible (6 patients). Other exclusion criteria were the infeasibility of an ultrasound examination because of medical reasons or because an ultrasound examination would have led to an unacceptable delay in therapy.

Patients’ age, height, weight and Body Mass Index (BMI), as well as liver-specific laboratory data (alanine aminotransferase (ALAT), aspartate aminotransferase (ASAT), gamma-glutamyl transferase (GGT), bilirubin and platelet count) were collected from the electronic database and correlated with the volumetric results. The thickness of subcutaneous fat tissue in CT was measured in each patient, correlating to the region in which the ultrasound was performed. Fibrosis-4 score (FIB-4) was determined in order to draw conclusions about the degree of fibrosis of the liver^16^ as follows^17^:

$${\text{FIB}} - {\text{4 Score }} = \, \left( {{\text{Age}}*{\text{AST}}} \right) \, / \, \left( {{\text{Platelets}}*\surd \left( {{\text{ALT}}} \right)} \right).$$

### Image acquisition

**Figure 1:**
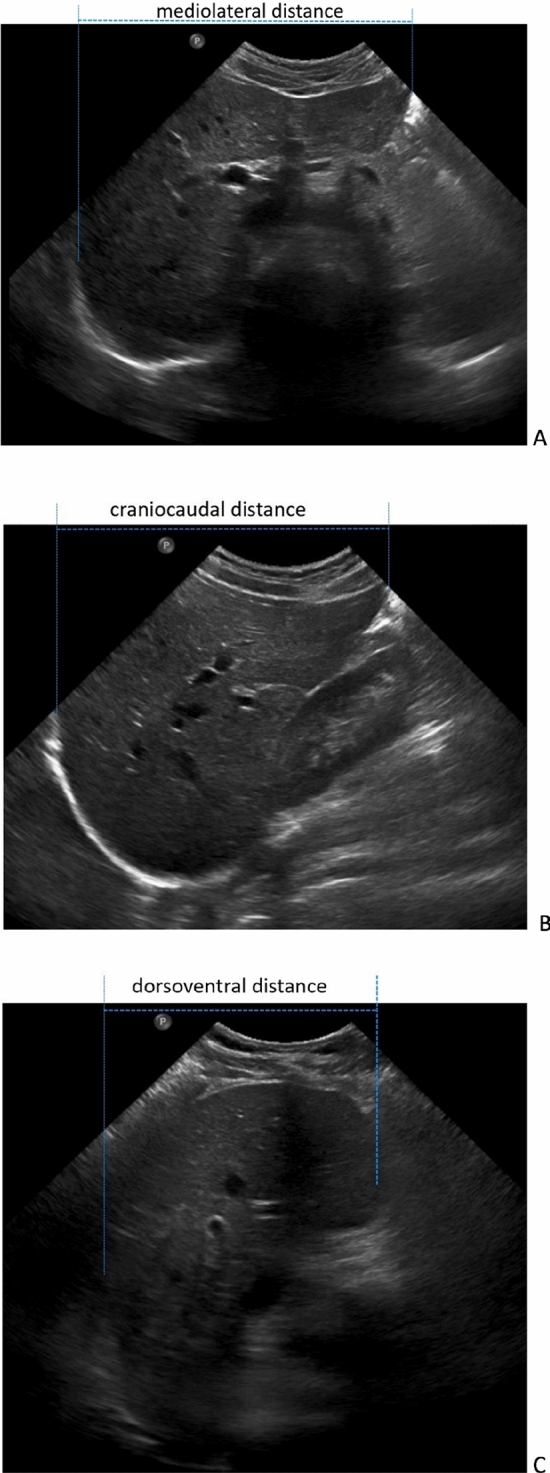
Example of a representative measurement of diameters in ultrasonography. Images were taken during the measurement of maximum diameters in (**A**) mediolateral (USmaxML), (**B**) craniocaudal (USmaxCC) and (**C**) dorsoventral direction (USmaxDV).

The ultrasound examination was carried out directly before CT scan and thus before application of oral contrast agent. Ultrasound examinations were performed with a convex transducer (5 GHz) of the ultrasonic device (Phillips Affiniti 50G, Philips Healthcare, Germany) in supine position by a trained radiology resident with 4 years experience. Additionally, 10 patients were examined by a trained specialist for internal medicine with 2 years of experience in ultrasonography. Both examiners were blinded to each other's measurements and to the results of the CT examination. Image acquisition was performed using subcostal and intercostal positions of the ultrasound probe. The measurements were standardized in strictly sagittal, coronal and transverse orientation to ensure the best possible comparability with the planes reconstructed in CT. Within the selected plane, the liver was fanned out in a structured manner by tilting and sliding while avoiding rotation of the ultrasound probe. Several measurements were taken within each orientation to ensure that the longest diameter could be determined at the level of the maximum transverse (mediolateral, USmaxML), sagittal (dorsoventral, USmaxDV) and coronal (craniocaudal, USmaxCC) diameter in all patients (Fig. 1).

In cases where a diameter could not be measured in a single ultrasound window, the measurements within one plane were combined using anatomical landmarks (portal vein or hepatic vein) and the measured diameters were added together. In all three dimensions, the borders of the liver had to be clearly visible. All measurements were carried out during apnea at the end of deep inspiration of the patient. This simplified reproducible detection of anatomic structures for the investigator and maintained the position for the patient during the measurement.

Volume indices were calculated following the description above, using the formula:

$${\text{Volume index }}\left( {{\text{US}}} \right) \, = \, \left( {{\text{USmaxML }} \times {\text{ USmaxDV }} \times {\text{ USmaxCC}}} \right) \, /{ 3}.{6}$$

as described by Roloff et al.^15^.

**Figure 2:**
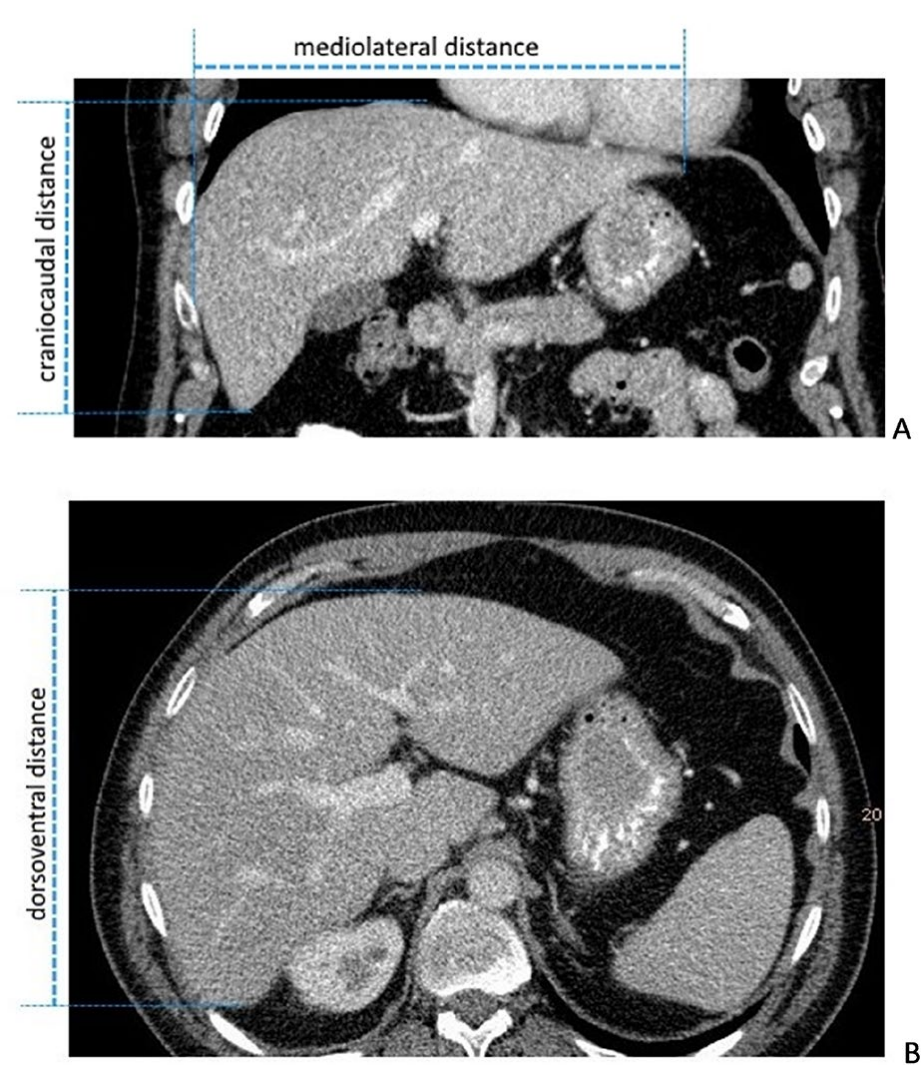
Representative measurement of maximum diameters in CT. Measurements were taken in the three standard dimensions (**A**) mediolateral (CTmaxML) + craniocaudal (CTmaxCC) and (**B**) dorsoventral (CTmaxDV).

CT examinations were performed with a 128-slice CT scanner (Somatom Definition AS + , Siemens Healthcare, Germany) with a collimation of 128 × 0.6 mm and a gantry rotation time of 0.28 s. CT data were acquired in the caudocranial direction and within one breath hold. In all examinations, the liver was completely covered, regardless of whether intravenous contrast medium administration was necessary or in which contrast medium phase the liver was examined.. Image reconstruction was done in transverse slices with a thickness of 3 mm. Measurements of CT diameters were performed using a picture archiving and communication system (PACS, IMPAX EE R 20, Agfa Healthcare, Mortsel, Belgium). In CT axial slices and coronal reconstructions were used to measure the maximum diameter in the mediolateral (CTmaxML), dorsoventral (CTmaxDV) and craniocaudal (CTmaxCC) direction by two trained observers, one radiologist with more than 4 years of experience in abdominal imaging and a trained medical student (Fig. 2), respectively. Both evaluators performed the reading independently and were blinded to each other`s results as well as to the results from the ultrasound. Thereafter, we measured the volume index based on CT diameters as follows:

$${\text{Volume index }}\left( {{\text{CT}}} \right) \, = \, \left( {{\text{CTmaxML }} \times {\text{ CTmaxDV }} \times {\text{ CTmaxCC}}} \right) \, /{ 3}.{6}$$

as described by Roloff et al.^15^.

### Standard of reference

**Figure 3:**
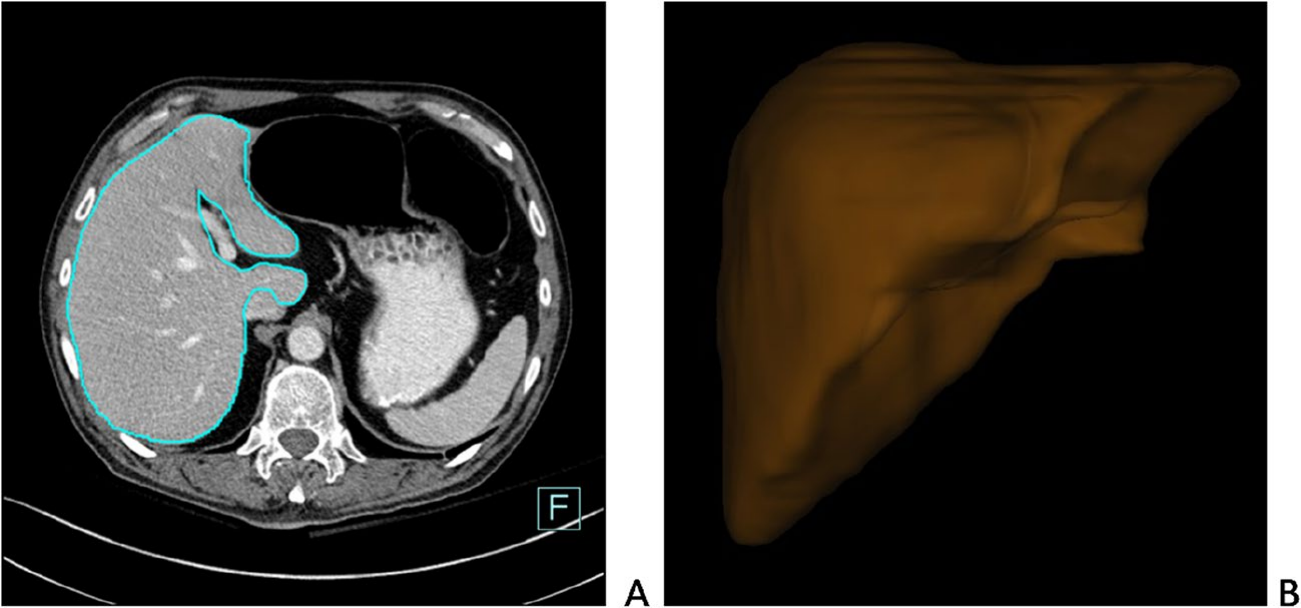
Refined contour of the liver after semi-automatic extraction of the liver parenchyma from abdominal CT-image in Syngo.Via and excluding the hepatic and portal vein (**A**) 3D model of the liver after post-processed liver shape (**B**).

Liver segmentation following the assessment of the total liver volume was defined as gold standard. It was performed in transverse CT slices with a thickness of 3 mm. Quantitative analysis was carried out with a semiautomatic volumetric program (Siemens Syngo.Via Multimodality Workplace; Version VB30A_HF01, Siemens, Germany) to segment the liver from the surrounding tissue by using HU-based thresholding. After pre-processing for complete liver segmentation, manual corrections for the determination of the contour of the liver were done if necessary and big vessels including the portal and hepatic vein were excluded (Fig. 3). The segmentation was performed by the same readers that measured liver diameters. They were independent of each other and blinded to the results of diameter measurement, ultrasound examination and clinical data.

### Statistical analysis

Maximum diameters measured in US and CT were presented as means and standard deviations. Quality management was performed via double reading by two observers of diameters in US in 10, and CT in all patients. For inter-rater reliability mean differences in percentages and standard deviations were calculated. Intraclass correlation for inter-rater variability was performed to compare VI and the diameter determinations in the different planes both in CT and in US.

The maximum diameters measured in US and CT were compared using Bland–Altman analysis referenced to the CT volumetry as gold standard. Mean bias and corresponding 95% confidence intervals (CI) were calculated^18^.

The volume indices from US and CT were analyzed in comparison to liver volume from segmentation with linear regression, and coefficient of determination (r^2^) was calculated. The correlation coefficient was interpreted according to Evans' classification as very weak (r^2^ = 0.00–0.19), weak (r^2^ = 0.20–0.39), moderate (r^2^ = 0.40–0.59), strong (r^2^ = 0.60–0.79) and very strong (r^2^ = 0.80–1.00)^19^. In addition, we performed Bland–Altman analysis to compare volume index of US, respectively CT with the gold standard.

Multivariable linear regression was applied for comparison of the calculated and the true liver volume in 59 patients. Therefore, we used the difference between the calculated and the true liver volume as outcome and age, sex, BMI, thickness of the subcutaneous fat layer, laboratory data and the Fib-4 score as potential predictor^20^. A backward selection algorithm was applied keeping only predictors with a *p* < 0.1. as potential predictors.

All evaluations were done with IBM SPSS Statistics 25 (IBM Corporation, Armonk, NY).

## Results

The inter-rater reliability of VI based on diameter measurements using US showed a mean difference of − 34.7% (CI − 90.1, 20.7%). The highest agreement between the two raters was found in the measurements of the diameter in the mediolateral direction (− 2.7%; CI − 29.8, 24.4%), whereas the dorsoventral (− 13.3%; CI − 32.0, 5.4%) and craniocaudal (− 19.5%; CI − 45.7, 6.8%) orientation showed a much higher deviation. In contrast, there was an excellent inter-rater reliability of VI based on CT diameters showing a low mean difference of 1.1% (CI − 16.1, 18.2%). CT diameters revealed the smallest deviations between the raters for the mediolateral direction (2.3%; CI − 16.5, 21.1%) and a higher deviation in the dorsoventral (3.2%; CI − 9.2, 15.7%) and craniocaudal (− 5.3%; CI − 25.2, 14.6%) orientation.

**Figure 4:**
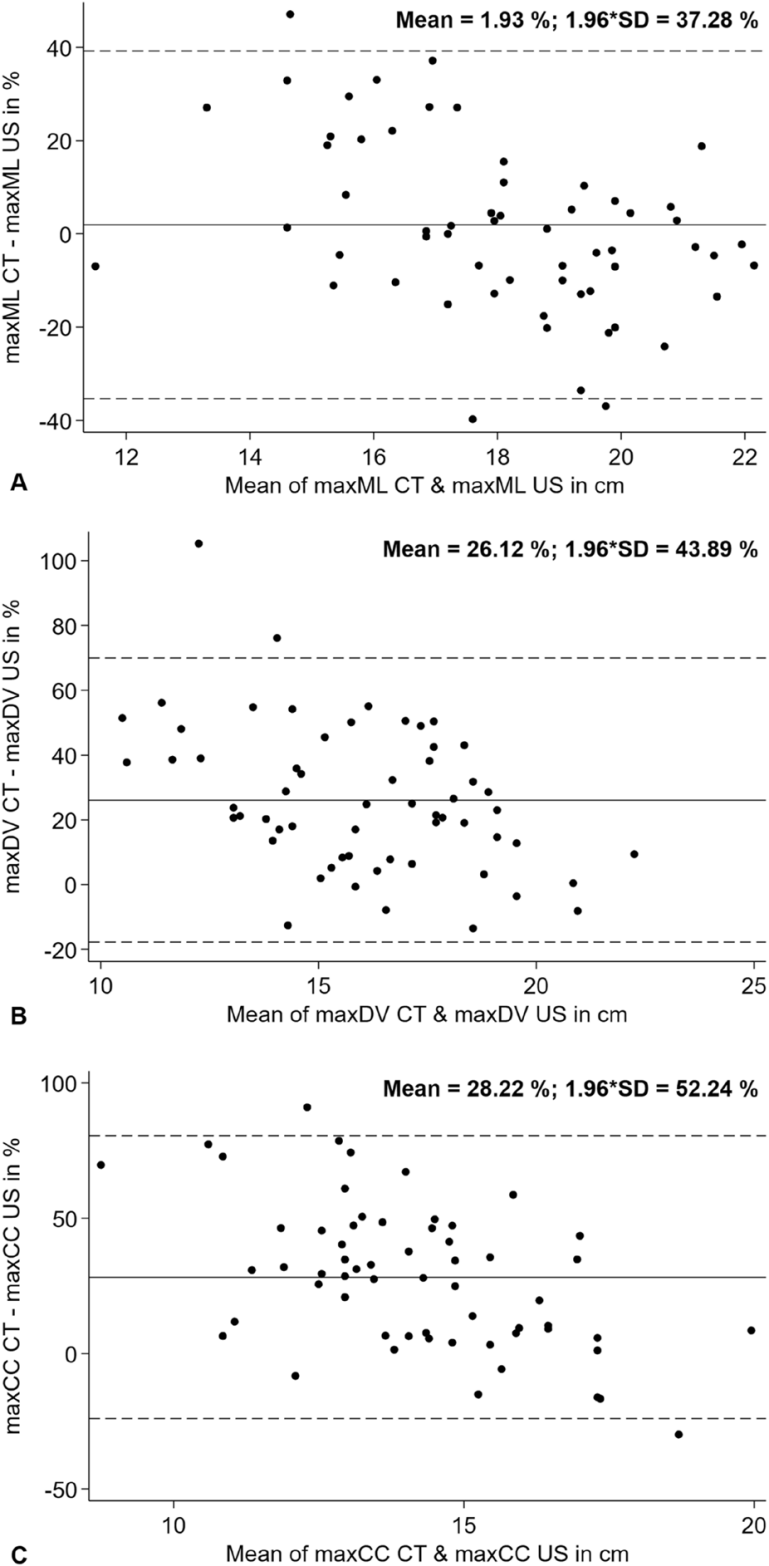
Bland–Altman correlation between the diameters measured in maximum extension in ultrasound and CT (**A**) in mediolateral, (**B**) dorsoventral and (**C**) craniocaudal orientation with showing the smallest difference in mediolateral extension.

Bland–Altman analysis showed a moderate agreement between the maximum diameters measured in ultrasound and CT (Fig. 4). The highest correlation was found in mediolateral orientation and the lowest in craniocaudal direction. Mean values and standard deviation are shown in Table 1.

**Table 1:** Mean and standard deviations of maximum diameters measured in ultrasound, computed tomography and true liver volume extracted from manual liver segmentation.

	Ultrasonography	Computed tomography	True liver volume
maxML(cm)	18.2 ± 3.4	18.2 ± 2.4	
maxDV(cm)	13.9 ± 3.4	17.9 ± 2.7	
maxCC(cm)	12.3 ± 2.9	16.0 ± 2.1	
Mean liver volume (cm^3^)	863.4 ± 371.8	1454.7 ± 414.4	1500 ± 347.8

The mean difference was low for the mediolateral direction (–0.6 cm; CI − 6.7, 5.6 cm), and larger (underestimated) for the dorsoventral (–4.1 cm; CI − 10.3, 2.1 cm) and craniocaudal direction (–3.6 cm; CI − 10.5, 3.3 cm). The results of the intraclass correlation are summarized in Table 2.

**Table 2:** Intraclass correlation coefficients for inter-rater variability.

	Volume index	Craniocaudal diameter	Dorsoventral diameter	Mediolateral diameter
CT	0.94	0.88	0.96	0.86
US	0.29	0.48	0.76	0.72

**Figure 5:**
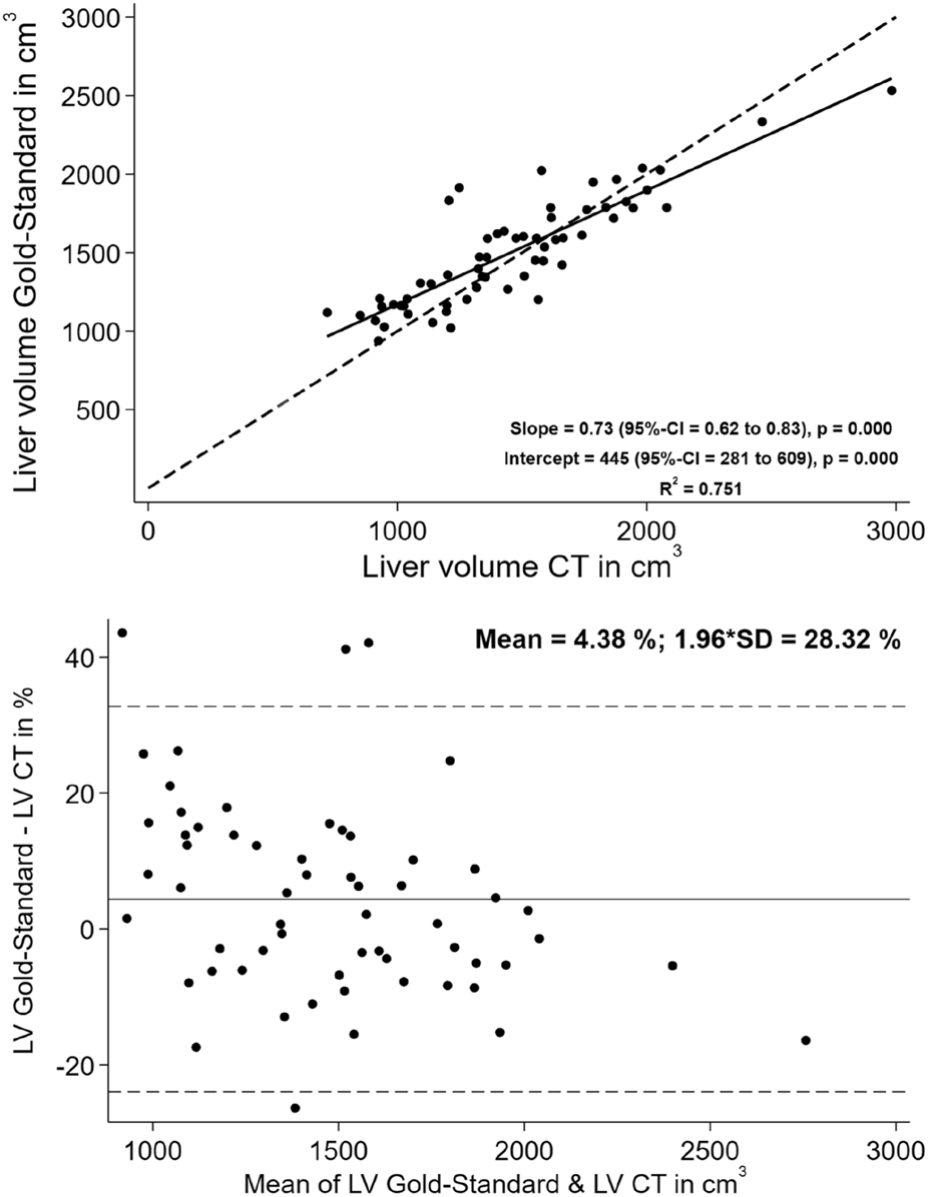
Linear correlation and Bland–Altman correlation between calculated VI from CT and true liver volume.

Using CT volumetry, the average total liver volume was measured as 1.500 ± 347 cm^3^, showing a strong correlation between the volume indices calculated from diameter measurement and the true liver volume (r^2^ = 0.751, Fig. 5). With 4.4 ± 28.3% (Fig. 5) (1.509 ± 432cm^3^ vs. 1.500 ± 347cm^3^) the mean bias was small. The largest differences between the results of volumetry and those of VI were found in patients with significantly enlarged right lobe or pronounced liver parenchyma changes.

There is a strong correlation (r^2^ = 0.751) between the calculated volume indices from maximum diameters measured in computed tomography (LV CT) and the true liver volume extracted from manual segmentation. There is only a small bias between the calculated volume indices from maximum diameters measured in CT (LV CT) and the true liver volume extracted from manual segmentation.

**Figure 6:**
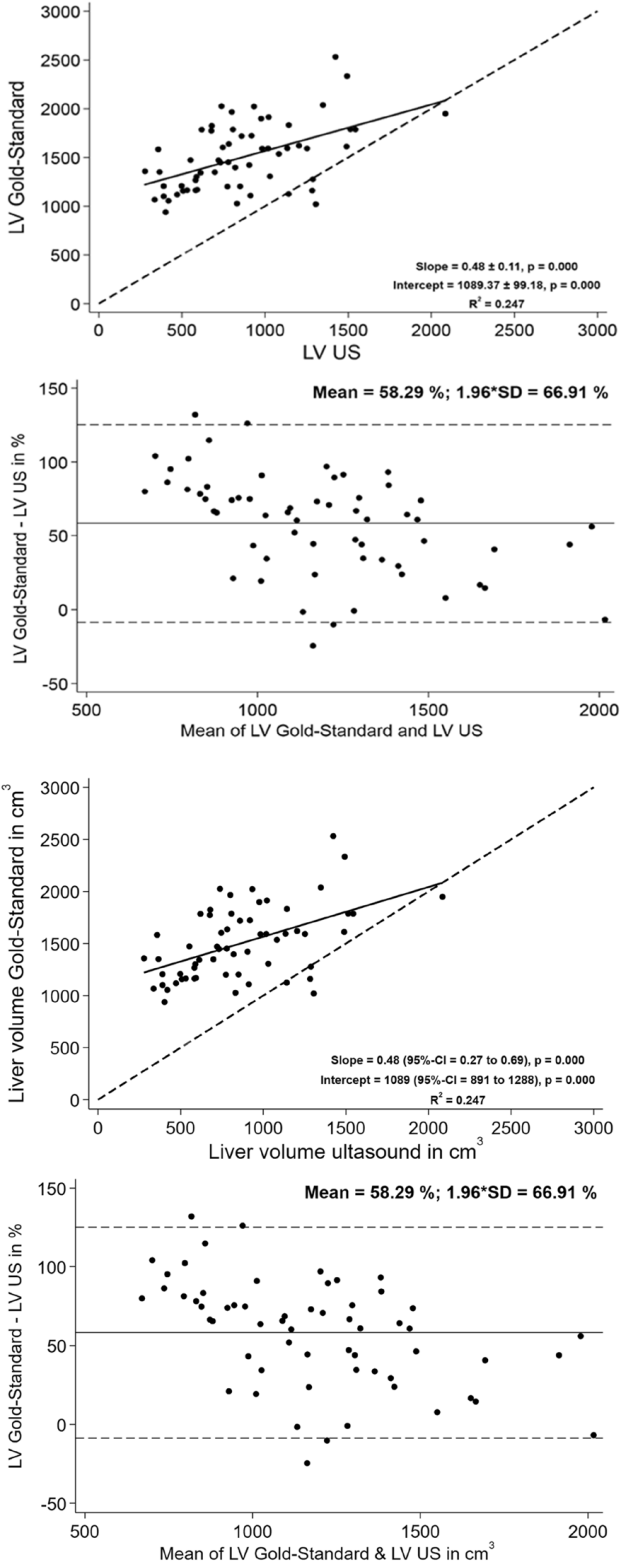
Linear correlation and Bland–Altman correlation between calculated VI from US and true liver volume.

In contrast, US revealed an average total volume of the liver of 863 ± 371 cm^3^ with a weak correlation between the calculated liver volume from US and the true liver volume (r^2^ = 0.247, Fig. 6). In comparison to true liver volume, measurement of liver volume using US demonstrated a mean difference of 58.29 ± 66.91% (Fig. 6). Thus, the total volume from ultrasound underestimates the true liver volume severely.

There is only weak correlation between the calculated volume indices from maximum diameters measured in ultrasound (LV US) and the true liver volume extracted from manual segmentation. A high bias was seen between the calculated volume indices from maximum diameters measured in CT (LV CT) compared to the true liver volume extracted from manual segmentation.

Correlation analysis did not show any dependency between the actual liver size and body mass index, the measured thickness of subcutaneous fat or the different laboratory values. However, there was a correlation between an increased Fib-4 Score and a lowered inter-rater agreement of the measured diameters in the CT (β = 59.1, 39.8–78.4; *p* < 0.001). The patients’ characteristics and laboratory data are summarized in Table 3.

**Table 3:** Patients characteristics and laboratory data.

Patients characteristics / Laboratory data	Mean ± standard deviation
Age	66.8 ± 12.6 years
Height	1.71 ± 0.1 m
Weight	79.8 ± 17.6 years
Body mass index	26.9 ± 5 kg/m^2^
thickness of subcutaneous layer from ventral	17.0 ± 8.9 cm
thickness of subcutaneous layer from lateral	12.7 ± 8.1 cm
ALAT	0.44 ± 0.25 µmol/(s*L)
ASAT	0.55 ± 0.54 µmol/(s*L)
GGT	1.08 ± 2.04 µmol/(s*L)
Bilirubin	9.01 ± 4.93 µmol/L
Platelet count	227.33 ± 88.96
Fib-4 score	2.11 ± 1.6 (N = 23 < 1.45, N = 7 > 3.75 and N = 24 in between)

## Discussion

To the best of our knowledge, our study is the first, which investigated the accuracy of a simple technique to estimate liver volume by the calculation of VI on the basis of routine US and CT examination data. The results of our study showed that only the calculated volume indices based on diameter measurements derived from CT are a valid approach for the estimation of liver volume, which underlines previous research^15^.

Determination of liver size using simple and reliable techniques is clinically warranted. Different approaches to asses liver volume in ultrasound have been presented based on the measurement of liver diameters in one^21–23^ and more than one dimensions^24^. In our opinion, the determination of the liver volume based on the measurement in one plane is questionable due to the high susceptibility to errors caused by possible anatomical variations or potential measurement inaccuracies. In a clinical setting, it is accepted that liver volume is enlarged if the craniocaudal distance measured by US in the midclavicular line exceeds 16 cm^21^. This observation must be critically questioned since no results for reliability were reported and our data clearly show a poor inter-rater agreement for diameter measurement using ultrasound. However, our own data demonstrated high inter-rater repeatability of CT diameters. Therefore, the estimation of liver volume using one dimension is possible if the diameter is reliably assessed, for example using cross-sectional imaging. This fact needs to be further explored.

The calculation of the volume index to predict liver volume is another simple approach based on the measurement of liver diameters in more than one dimension. Various formulas for estimating the liver volume using volume indices have been presented so far. An approach by Boscaini et al. used the product of measuring three diameters (length, width, height) and divided this by 27^25^. This approach saw the liver in the form of a cube, which explains the lack of accuracy. A further development of this approach was provided by Marchesini et al.^26^ and Zoli et al.^27^ by comparing volume calculations with CT-based volume determinations. However, the authors described difficulties in comparability and therefore proposed a calibration to eliminate them. Muggli et al.^28^ followed an approach to determine liver volume on the basis of diametric measurements, however, patients with changes in liver parenchyma were excluded. For this reason, the results appear to show clear limitations for clinical practice. These could also be the reason why the results of the calculated VI appear slightly better in comparison to the actual volume than in our study.

This assumption is supported by the fact that our results showed a decreased interobserver agreement in the calculated VI in patients with an increased fib-4 score, which explains a lower accuracy of VI in presents of liver parenchyma changes.

In previous research, a calibration of volume index determined by the three diameters in their maximum orientation divided by the factor 3.6 was introduced. Using this factor, the volume index and the true liver volume are comparable. However, an accurate estimation of liver volume using volume indices requires also a reliable and robust assessment of liver diameters. As shown in our study results, we found excellent inter-rater reliability if liver diameters are determined in CT images, but not for US. This is also in line with the results from Verma et al., which showed good inter-rater reliability for measurements of diameters using cross-sectional imaging like MRI and a good correlation to hepatic volume^29^. In addition, the result seems plausible, since in CT the measurements are based on an identical data set, whereas in sonography the measurements of the two raters are based on different images that they acquire themselves.

It must be critically noted that the variability of the differences between VI from CT and volumetry is relatively high, which can be attributed to the fact that volumetry based on segmentation is more adaptable to anatomical variabilities of the liver than the method of diameter measurement. This has to be considered in particular against the background that the highest deviations between volumetry and VI were found in patients who showed a significant enlargement of the right hepatic lobe (riedel’s lobe^30^) or pronounced changes of the liver parenchyma as in liver cirrhosis.

A further clear advantage of volumetry by segmentation is that it can also be performed for only partial areas of the liver, which plays an important role in preoperative planning prior to liver resection. Good results in the agreement of the volumetrically determined volume both in MRI and CT with the actual postoperative liver volume could be shown in the work of Karlo et al.^31^. These partial volume measurements are clearly limited when determining the liver volume on the basis of VI.

Our study results further demonstrated a worse agreement of liver diameters if they were assessed using US. We could exclude possible objective reasons for that, such as patient´s body constitution (investigated by body mass index and size of the subcutaneous fat layer) as well as variations of the liver itself such as parenchymal liver diseases (investigated by liver volume, Fib4-score, laboratory data). Using our correlation, we cannot clarify for sure the reason for the lower inter-rater reliability for measurements of diameters using US with the exception of the methodological influences such as the cooperation of the patient during inspiration or the measurement inaccuracies resulting from the different posture of the ultrasound probe.

This is supported in particular by the fact that a significantly poorer correlation can be seen in measurements within the craniocaudal plane. In addition to the fact that the ultrasound conditions were most clearly impaired in this plane due to anatomical features such as the costal arch and the resulting restricted freedom of movement of the ultrasound transducer, this can be explained in particular by the respiration-dependent changes, particularly in this plane, and the associated changes in the position of the liver, lungs and diaphragm. In the other two dimensions, there is a much better correlation between the raters, indicating that the patient's influence on the measurements is less pronounced. In addition, a good and almost good correlation between the raters in dorsoventral and mediolateral suggests a proper training.

A further potential source of error can be suspected in cases where the diameters were not determined within one position of the ultrasonic probe but had to be calculated on the basis of two positions. In this case, despite the greatest care, it cannot be guaranteed that the second setting will exactly match the plane of the first setting, which is an additional source of error.

A possible improvement approach in the future could be the use of 3D ultrasound. This method, which is widely used in gynecological imaging^32^, does not yet play a clinical role in imaging the liver. However, there are already first diagnostic approaches to use it for imaging the liver surface in parenchymatous diseases^33^ and for monitoring during interventional procedures^34,35^. Regarding correlation of liver measurements with clinical parameters, we found only a correlation between the FIB-4 and the inter-rater reliability in the diameter measurement in CT. Among other things, this could be due to the change of the liver surface, which can lead to deviations in the measurements due to the difficulty in determining the exact maximum diameter in case of irregularities of the liver surface in cirrhosis.

Consistently, our study shows a significant poorer inter-rater reliability in US compared to CT and results further suggest that the volume of the liver using US-based calculated VI does not lead to a valid result. In our opinion, calculation of volume index based on measurement of liver diameters using CT is an excellent, simple approach to predict liver volume. This simple technique is ready for clinical setting.

There are some limitations in our study. Study patients underwent CT exclusively for clinical reasons. As such, a clinically healthy control group was missing for comparison. However, in previous research, the excellent accuracy of calibrated volume indices to estimate liver volume in a cohort of volunteers^15^ was confirmed. In this study, we intentionally focus on a patient cohort to demonstrate that our approach is applicable in clinical practice. A further limitation of the study is that among the subjects included, no one had previously undergone liver surgery. A statement regarding the value of VI, for example after hemihepatectomy, is still to be investigated. In addition, it is a known obstacle in ultrasound to obtain exact orientations within a plane. Nevertheless, despite rigorous efforts (multiple control of the measurement level) and extensive previous training, there may have been measurement inaccuracies by distortions, which may have influenced the results. Furthermore, the level of experience of the examiners, especially in such an examiner-dependent procedure as ultrasound, must be considered as a possible cause for the low inter-rater agreement.

In conclusion, the volume index is an excellent approach to estimate liver volume very fast using a routinely available data set. However, an accurate estimation of liver volume requires a robust and reliable assessment of liver diameters as provided by cross-sectional imaging, such as CT. US cannot reliably measure maximum liver diameters, wherefore the usage of US-based volume indices should not be used due to its low accuracy.

## Data Availability

The datasets used and analysed during the current study are available from the corresponding author on reasonable request.
